# Anticoagulant Overdose With Paradoxical Thrombotic Risk

**DOI:** 10.7759/cureus.76116

**Published:** 2024-12-21

**Authors:** Ramanathan Moorthy, Kush Deshpande

**Affiliations:** 1 Intensive Care Medicine, St George Hospital, Sydney, AUS; 2 Intensive Care Medicine, University of New South Wales, Sydney, AUS

**Keywords:** metallic heart valve, noncompliant with anticoagulation, paracetamol overdose, prothrombotic vs bleeding risk, warfarin overdose

## Abstract

We report a case of an unusual polypharmacy overdose including warfarin in a patient with a metallic heart valve, complicated by the history that he had ceased taking anticoagulation in the preceding few months, that placed him in an initial prothrombotic phase during his presentation. Our case highlights the importance of elucidating all relevant clinical details in a polypharmacy overdose, as clearly and rapidly as feasible, to enable appropriate management.

## Introduction

Presentations involving polypharmacy overdose are often challenging due to the varied adverse effects of the agents that a person may have taken. The toxicological effects can sometimes have an exaggerated impact on an individual’s overall systemic stability, based on the past medical and surgical background [1]. To add more complexity to these scenarios, if a reliable clinical history is not attainable due to the patients being intoxicated, the challenges faced by the treating teams are multiplied many folds [2,3]. Warfarin overdose is rare, as evidenced by a retrospective study over 25 years that showed only 23 cases [4]. Furthermore, we could not find any publication that described a warfarin overdose in a patient with a metallic heart valve, who had ceased taking warfarin prior to the overdose. We discuss the clinical course of one such patient who was managed in our institute.

## Case presentation

We report the case of a 64-year-old male, who lived alone and was brought to our hospital on an evening following polypharmacy overdose due to suicidal ideation. His personal stressors involved flooding of his home, difficult neighbors, loss of job, and the recent death of his partner. Fearing an impending eviction from his property, he had mentioned to his property agent that he would end his life, prompting a police welfare check resulting in his hospitalization. He had allegedly crushed some of his available regular medications and consumed about 10g of paracetamol, unknown amounts of telmisartan, metoprolol, and possibly warfarin, along with a bottle of wine. The exact timing and quantities consumed were not known due to his intoxicated state and inadequate circumstantial evidence at his home. He had likely taken the overdose about two to three hours prior to his arrival. He had a history of hypertension, coronary artery bypass surgery, and an aortic valve replacement with a metallic valve.

On arrival at the emergency department (ED), he was hemodynamically stable with an audible metallic click on cardiovascular examination but in an intoxicated state with intermittent agitation. Initial attempts by emergency and mental health teams to elucidate all relevant history were not successful. His blood results on admission showed mildly raised creatinine, a normal coagulation profile, and an elevated paracetamol level that did not need therapy with n-acetylcysteine as per toxicological advice based on the nomogram. He required a few doses of diazepam in the initial few hours after arrival to help with alcohol withdrawal. After a few hours of observation in the ED, he was admitted to the intensive care unit (ICU) for ongoing clinical management, including tests to check serial paracetamol levels and INR (international normalized ratio) among others. His repeat INR was 1.2 about eight hours after presentation, and his paracetamol level was declining. His alanine transaminase level remained normal, and his serum creatinine level was improving. He had slept overnight in the ICU without any problems. He remained hemodynamically stable, suggesting that the quantities of metoprolol and telmisartan consumed were likely insignificant.

Upon waking up the next morning, he regretted his act without any ongoing suicidality. He mentioned that he had ceased taking warfarin a few months back due to fear of adverse effects but that he may have taken between 12 to 14 mg of warfarin the preceding day. He said that he practiced taking 1-2 liters of tomato and watermelon juice daily, as he believed that this could help him remain healthy.

The above history led to the recognition of the initial prothrombotic phase that he was going through, prompting therapeutic heparin anticoagulation to mitigate this risk, as his INR values till then were normal corroborating his history of having ceased warfarin. He understood the high risk that he was facing due to his overdose and consented to comply with all medical advice. Urgent discussions with both hematology and cardiology teams supported this decision at this juncture. On review of events since admission, it became clear that adequate clarification regarding his preceding cessation of warfarin and the current overdose including this was not achievable until he was awake.

Heparin anticoagulation was ceased once the INR reached 2.5. His subsequent daily INR values incremented as expected due to the ongoing effect of warfarin. As his INR level increased beyond 10 three days after presentation, he received a dose of intravenous (IV) vitamin K 2 mg, which corrected it to 2.2. He did not have any bleeding and remained stable. He was cleared by the mental health team as a case of an overdose due to a situational crisis, without ongoing suicidality or need for detention.

His serial relevant blood test results and the paracetamol level are shown in Table 1.

**Table 1:** Serial laboratory results Paracetamol level: critical level at four hours post ingestion is 150 mg/L. Ethanol level: 11 mmol/L is equivalent to 0.05% (legal limit for normal driving license holders in New South Wales, Australia) INR: normal range 0.8 to 1.3. Range of 2.0-3.0 is recommended for the management of venous thromboembolism, prevention of stroke in non-valvular atrial fibrillation, and low-risk prosthetic heart valves. Higher INR values are required in higher risk prosthetic heart valves (target 3-3.5) and recurrent thromboembolism. APTT: normal range is 25 seconds to 37 seconds ALT: normal is less than 51 U/L Creatinine: normal range is 60 micromole/L to 110 micromole/L Discharged on day 4 ALT, alanine transaminase; APTT, activated partial thromboplastin time; INR, international normalized ratio

Day/time	Paracetamol level (mg/L)	Ethanol level (mmol/L)	INR	APTT (seconds)	ALT U/L	Creatinine (micromole/L)
Day 0/18:41	30	46; 0.21%	1.0	-	31	155
Day 1/01:25	22	-	1.2	24.1	29	126
Day 1/09:29	7	-	1.7	30.1	24	111
Day 1/17:33	-	-	2.5	125.0	-	101
Day 2/06:34	-	-	4.9	34.0	20	85
Day 3/06:46	-	-	>10	41.1	30	92
Day 3/18:04	-	-	2.2	36.0	35	92
Day 4/09:25	-	-	2.9	42.2	35	92

## Discussion

We would like to highlight some interesting points in this case.

First, though warfarin overdose has been reported previously [4,5], it appears to be rare to see warfarin overdose in a patient with a metallic heart valve who had ceased taking warfarin. This high-risk combination seems rare.

Second, the specific combination of having a metallic heart valve but having ceased warfarin months prior to presentation poses the prothrombotic risk [6] during re-warfarinization, with the need for bridging anticoagulation to mitigate this risk [7,8]. Though warfarin inhibits activity of vitamin K-dependent clotting factors, it also inhibits anticoagulant factors, protein C, and protein S. As protein C has a shorter half-life (eight hours) than clotting factors, it is inhibited first with a resultant procoagulant state in the initial phase after warfarin administration [9]. Due to the uncommon nature of this situation, it is to be emphasized that inexperienced staff may potentially not acknowledge this initial prothrombotic risk, with potentially unpleasant consequences [10,11].

Third, as he had also taken an overdose of paracetamol, lack of INR elevation as evidence against paracetamol toxicity gave a sense of false reassurance of overall patient safety. On the contrary, our patient with a metallic heart valve was passing through an initial risky procoagulant phase needing alternative therapeutic anticoagulation.

Fourth, when his INR reached alarming levels (>10), it was not easy to dose vitamin K due to the risk of overcorrection with clotting risk. As our patient did not have bleeding concerns, he was given 2 mg intravenous vitamin K after discussion with the hematology team, with appropriate correction of his INR.

Lastly, our patient is similar to one of those rare cases who have a metallic heart valve but survived without any anticoagulation over a prolonged period [12,13]. Prosthetic aortic valves reportedly have lesser thromboembolic problems compared to mitral valves [14]. The antiplatelet and antithrombotic effects of tomatoes and watermelons have been described previously [15-17]. Our patient was consuming large quantities of these daily, but if these can substitute anticoagulation remains unclear.

Our patient was discharged in a stable condition, with follow-up by “hospital in the home” services, helping him titrate the warfarin dose to achieve a steady state. He was provided with resources to contact if he had any further concerns.

## Conclusions

Our case highlights the importance of relevant history of events in an overdose scenario, especially involving an uncommon agent such as warfarin and the need to be aware of the concomitant risks. In our patient, any prothrombotic situation could result in the thrombosis of the metallic heart valve and reduction in the cardiac output, leading to his collapse. But once the initial procoagulant phase passed, the bleeding risk needed cautious correction. This confusing scenario was because the INR, a specific test for coagulation, was normal. Though it was reassuring against paracetamol toxicity, but it also indicated a procoagulant risk with a metallic heart valve. We highlight this uncommon challenge with the need to weigh the risks of thrombosis in the setting of a metallic heart valve versus bleeding due to ongoing warfarin effect from the overdose and the close monitoring required to cautiously manage the patient. Antiplatelet and antithrombotic effects of certain fruits such as tomatoes and watermelon need further research and understanding for reliable clinical use.
